# Er-Xian Decoction Stimulates Osteoblastic Differentiation of Bone Mesenchymal Stem Cells in Ovariectomized Mice and Its Gene Profile Analysis

**DOI:** 10.1155/2016/4079210

**Published:** 2016-03-15

**Authors:** Shufen Liu, Jianhua Huang, Jing Wang, Yongjian Zhao, Sheng Lu, Yongjun Wang, Qin Bian

**Affiliations:** ^1^Department of Orthopaedics & Traumatology, Longhua Hospital, Shanghai University of Traditional Chinese Medicine, No. 725 South Wan-ping Road, Shanghai 200032, China; ^2^Spine Research Institute, Shanghai University of Traditional Chinese Medicine, No. 725 South Wanping Road, Shanghai 200032, China; ^3^Institute of Integrated Traditional Chinese Medicine & Western Medicine, Huashan Hospital, Fudan University, No. 12 Middle Wulumuqi Road, Shanghai 200040, China

## Abstract

We studied the bone mesenchymal stem cells (bMSCs) and gene profiles regulated by* Er-Xian Decoction* (EXD), a traditional Chinese herbal formula widely used for postmenopausal osteoporosis treatment. Six-month-old female Imprinting Control Region mice that underwent ovariectomy were treated with EXD. After 3 months, bone mass was evaluated by *μ*CT and histological and immunohistochemical detection. The self-renewal and differentiation capacities of bMSCs were evaluated by colony-forming unit-fibroblastic, colony-forming unit-adipocyte, and alkaline phosphatase staining. In addition, the expression of 26991 genes of bMSCs* ex vivo* at 2 weeks after EXD-treatment or of bMSCs* in vitro* after exposure to conditioned serum from EXD-treated rats was measured and analyzed using NimbleGen Gene Expression Profiling and Cluster and pathway analysis. EXD treatment increased bone mass, elevating osteocalcin protein levels* in vivo* and facilitating the self-renewal and osteoblastic differentiation of bMSCs* ex vivo*. EXD rescued several gene expressions that were dysregulated by OVX. These genes overlapped and their functions were involved in ten pathways between* ex vivo* and* in vitro* experiments. EXD exerts an osteogenic effect on bMSCs in OVX induced osteoporotic mice. Our results contribute to further study of its molecular mechanism and traditional use in the treatment of postmenopausal osteoporosis.

## 1. Introduction

*Er-Xian Decoction* (EXD), a traditional Chinese herbal formula, has been clinically used in relieving menopausal syndrome such as postmenopausal osteoporosis. A meta-analysis of 5 studies including 677 participants and a recent randomized, double-blind, controlled trial demonstrated that EXD is effective in treating menopausal symptoms [1, 2]. The animal experimental data also showed an antiosteoporotic effect of EXD in ovariectomized (OVX) rats, which was involved in correcting the increased body weight, serum beta-glycerophosphatase and alkaline phosphatase while reserving the decreased bone mass density, biomechanical strength, concentration of calcium, phosphorus, and estradiol in serum [3, 4]. Importantly, EXD was found to have superior efficacy and safety profile than a single component [5, 6]. In addition, EXD modulates osteoblastic and osteoclastic activity in both* in vivo* and* in vitro* study [7, 8]. However, the effects of EXD on bone mesenchymal stem cells (bMSCs) and the molecular target of EXD in antiosteoporotic activity have not been fully elucidated.

BMSCs are the major source of osteoblasts, which play a key role in osteogenesis required for maintaining bone metabolic equilibrium. Of postmenopausal women, the osteogenic potential of bMSCs was lower than compared to healthy women [9]. Since EXD promotes osteoblastic proliferation as reported [8], we hypothesized that EXD exerts an osteogenic effect and promotes bMSCs activity in OVX-induced osteoporotic mice.

In the present study, we evaluated the osteogenic effect of EXD treatment in OVX mice using bone densitometry and 2D and 3D bone histomorphometries and histological and immunohistochemical detection. We evaluated the effect of EXD on the self-renewal and differentiation capacity of bMSCs by performing colony-forming unit-fibroblastic (CFU-F), colony-forming unit-adipocyte (CFU-Adipo), and alkaline phosphatase (ALP) assays. Furthermore, the EXD's molecular targets of bMSCs were evaluated in both* ex vivo* and* in vitro* experiments by using gene profile analysis. We illustrated the gene expression patterns* ex vivo* and demonstrated the overlapped pathways induced by EXD between* ex vivo* and* in vitro* experiments. Our results contribute to further study of EXD's molecular mechanism and traditional use in the treatment of postmenopausal osteoporosis.

## 2. Materials and Methods

### 2.1. Preparation for EXD

An EXD is composed of* Curculigo orchioides Gaertn* (9 g),* Herbaa Epimedii* (9 g),* Radix Morindae Officinalis* (9 g),* Radix Angelicae Sinensis* (9 g),* Cortex Phellodendri* (6 g), and* Rhizoma Anemarrhenae* (6 g). These herbs were purchased from Tian-jiang Pharmaceuticals Company (Jiangsu, CN). Five times the weight of EXD was added with 5 times distilled water. After soaked for 2 h, the mix was decocted twice, 2 h for each time, filtered, and concentrated to the final dose of 2.4 g/mL for the* in vivo* experiments. For the* in vitro* experiments, rats were intraperitoneally anesthetized 1 h after the last administration, and blood from the abdominal aorta was centrifuged to obtain EXD containing-serum. This conditioned serum was inactivated, filtered, and preserved at −70°C.

### 2.2. Animals Grouping and Treatments

Six-month-old female Imprinting Control Region (ICR) mice (*n* = 60) were provided by the Shanghai Laboratory Animal Center (SCXK 2007-0005, Science and Technology Commission of Shanghai Municipality; Shanghai Animal Ethics Committee approved for the experimental research on animals). The mice were randomly divided into sham, OVX, and OVX+EXD (EXD) groups. These groups were subdivided into 2 weeks and 3 months groups (*n* = 10 per group). The mice in EXD groups were intragastrically administered EXD at dose of 30 g/(kg·d) for 2 weeks or 3 months (twice every three days) at the 4th day after OVX surgery. Other 1-month-old male Sprague Dawley rats (*n* = 20) were intragastrically administered EXD 6 g/(kg·d) or physiological saline (PS, 1.25 mL/d) for three days to prepare for the conditioned serum used in an* in vitro* study.

### 2.3. *μ*CT

Lumbar spine specimens were fixed in 4% paraformaldehyde for 24 h, washed for 2 h, and examined. Six L_4_ vertebrae in each group were described for a 3D model without exhibiting adnexa, such as transverse and spinous processes. Analyses were performed using the *μ*CT 80 radiograph microtomograph (Scanco Medical AG, Switzerland), associated 3DCalc, cone reconstruction, and AVG model building software (HP, Japan). The scanner was set at a voltage of 55 kVp, a current of 72 *μ*A, and a resolution of 10 *μ*m per pixel. A reconstruction of the bitmap data set was obtained and employed to build the 3D model. Scores for the ratio of bone volume to tissue volume (BV/TV), the connectivity density of trabeculae (Conn.D.), the trabecular number (Tb.N), the trabecular thickness (Tb.Th), and the trabecular spaces (Tb.Sp) were directly obtained from the 3D model.

### 2.4. Histological and Histomorphometric Analyses

Lumbar spines were fixed in 4% paraformaldehyde for 24 h, decalcified in 10% EDTA for 2 weeks, and embedded in paraffin wax. The sections were stained with hematoxylin and eosin or following trap staining procedure. A morphometric study was performed with an image autoanalysis system (Olympus BX50; Japan). L_4_ were examined. The static parameters were the trabecular bone area (T.Ar) and the bone perimeter (B.Pm), which were utilized to calculate the parameters of bone formation: the activity of osteoblasts (N.ob/T.Ar, N.ob/B.pm) or the parameters of bone resorption: the activity of osteoclasts (N.oc/B.pm) [10].

### 2.5. Immunohistochemical and Immunofluorescence Analyses

Sections were pretreated and stained with osteocalcin (1 : 100, Abcam Ltd., Cambridge, UK), as previously described [11]. A morphometric study was performed with an image autoanalysis system (Olympus BX50, Japan). The data were quantified using a medical image management system (Cmias, CN).

### 2.6. BMSCs Culture and CFU-F, CFU-Adipo Assays

BMSCs were obtained from the bone marrow of the bilateral tibia and femur of ten mice per group in the 2-week-OVX experiment for microarray detection or in the 3-month-OVX experiment to evaluate the capacity of self-renewal and differentiation. The marrow cavity was flushed with alpha-MEM (Gibco, USA) containing 10% fetal bovine serum (FBS, Gibco, USA) and 1% penicillin-streptomycin (Gibco, USA), and grown in 10 cm plastic dishes (2 mice per dish). The number of spontaneously formed CFU-F or CFU-Adipo was counted under a light inverted microscope at 4th or 7th day. For* in vitro* study, the bMSCs were treated for 2 h with alpha-MEM containing 10% serum of rats that were pretreated with EXD or PS for three days (conditioned-serum).

### 2.7. ALP Assay

For the differentiation assay, bMSCs were cultured for 7 days, fixed with 10% formalin, and stained with NBT-BCIP (Pierce, USA) for 30 min. Lyons blue represents the positive staining. Quantitative analysis was conducted with Image-Pro Plus Software version 6.0 (Media Cybernetics Inc.). The percentage of positive staining was calculated by counting the positive staining in dish area. Fold changes were chosen for data display.

### 2.8. Oligo GEArray Experiments

Total 15 pieces of NimbleGen Gene Expression Profiling (number PXH100525) containing 26991 genes were used (3 pieces per group). Total RNAs of BMSCs are harvested using TRIzol (Invitrogen) and the RNeasy kit (Qiagen) according to manufacturer's instructions, including a DNase digestion step. After having passed RNA measurement on the Nanodrop ND-1000 and denaturing gel electrophoresis, the samples were amplified and labeled using a NimbleGen One-Color DNA Labeling Kit and hybridized in NimbleGen Hybridization System. After hybridization and washing, the processed slides were scanned with the Axon GenePix 4000B microarray scanner. Raw data were extracted by NimbleScan software (version 2.5). NimbleScan software's implementation of RMA offers quantile normalization and background correction. The gene summary files were imported into Agilent GeneSpring Software (version 11.0) for further analysis. Differentially expressed genes were identified through fold change and *t*-test screening. The profiling identified a subset of the total number of probes that are differentially expressed.

### 2.9. qPCR Detection

BMSCs collected from* ex vivo* experiment were directly processed following RNA preparation employing the PURE Prep Kit protocol. One microgram of total RNA was reverse transcribed using the Advantage RT-for-PCR Kit (Qiagen, Valencia, CA) following the manufacturer's protocol. Freshly transcribed cDNA (1 *μ*L) was employed for quantitative real-time PCR using SYBR Green (Bio-Bad, Hercules, CA) to monitor DNA synthesis with specific primers (Table 1) designed by NCBI/Primer-BLAST. Gene expression was normalized to the housekeeping gene *β*-actin. PCR products were subjected to melting-curve analysis, and the data were analyzed and quantified with the RotorGene 6.0 analysis software.

### 2.10. Statistical Analyses

The data are expressed as means ± SE, and statistical significance was calculated using One-Way ANOVA followed by a* post-hoc* LSD test (homogeneity of variance) or a Tukey's test (heterogeneity of variance) using SPSS software (SPSS Inc., Chicago, USA). The significance level was defined as *p* < 0.05. For microarray expression data analysis, pathway analysis was performed using the web-based DAVID DATABASE (http://david.abcc.ncifcrf.gov/) and achieved through functional analysis mapping of genes to KEGG pathways. Clustering analysis was performed with R software (version 2.11.1).

## 3. Results

### 3.1. *μ*CT 3D Morphometry of Bone Mass

**Figure 1:**
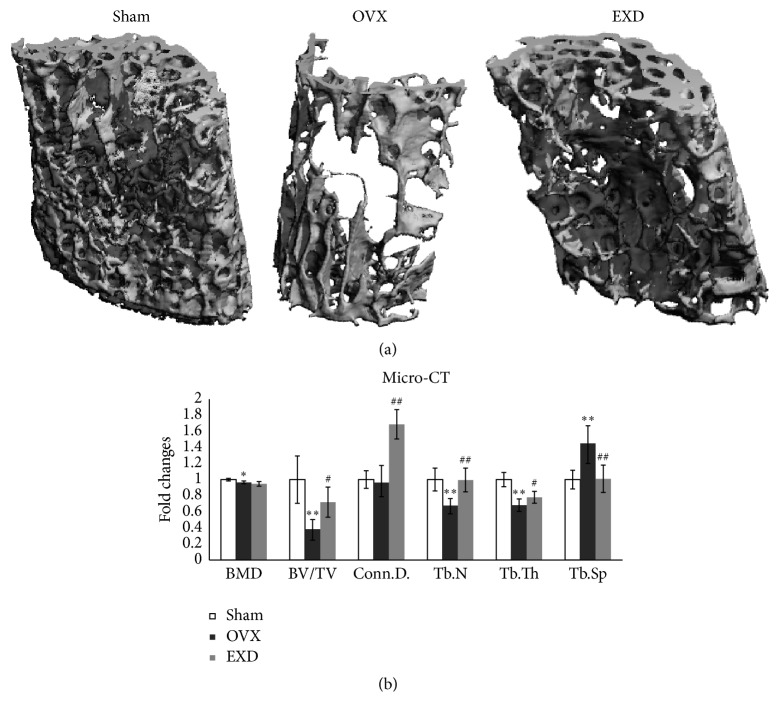
EXD treatment prevents OVX-induced bone loss by *μ*CT analyses. (a) 3D image of mouse L_4_ vertebra in Sham, OVX, and EXD group analyzed by *μ*CT. (b) Morphometric data of (a) indicates a bone loss by a significant decrease in BMD, BV/TV, Tb.N, Tb.Th and an increase in Tb.Sp. of OVX mice compared to those of Sham mice. Four of them were significantly rescued by EXD treatment. In addition, EXD markedly increased the Conn.D. degree after OVX surgery. The columns represent the means ± SE *n* = 6 per group. ^*∗*^ *p* < 0.05, ^*∗∗*^ *p* < 0.01 versus sham, ^#^ *p* < 0.05, ^##^ *p* < 0.01 versus OVX.

To study the antiosteoporotic effect of EXD, we employed a classic osteoporotic mouse model and tested bone density by *μ*CT. A significant decrease in BMD, BV/TV, Tb.N, and Tb.Th while an increase in Tb.Sp was observed at 3 months after the surgery as compared to the sham group, indicating a bone loss was induced by OVX. With EXD treatment, the BV/TV, Conn.D, Tb.N, and Tb.Th were significantly elevated as compared to the OVX group (*p* < 0.05 or *p* < 0.01). The value for Tb.Sp was also improved in EXD group (Figure 1).

### 3.2. 2D Morphometry of Osteoblasts and Osteoclasts, and OC Protein Distribution

**Figure 2:**
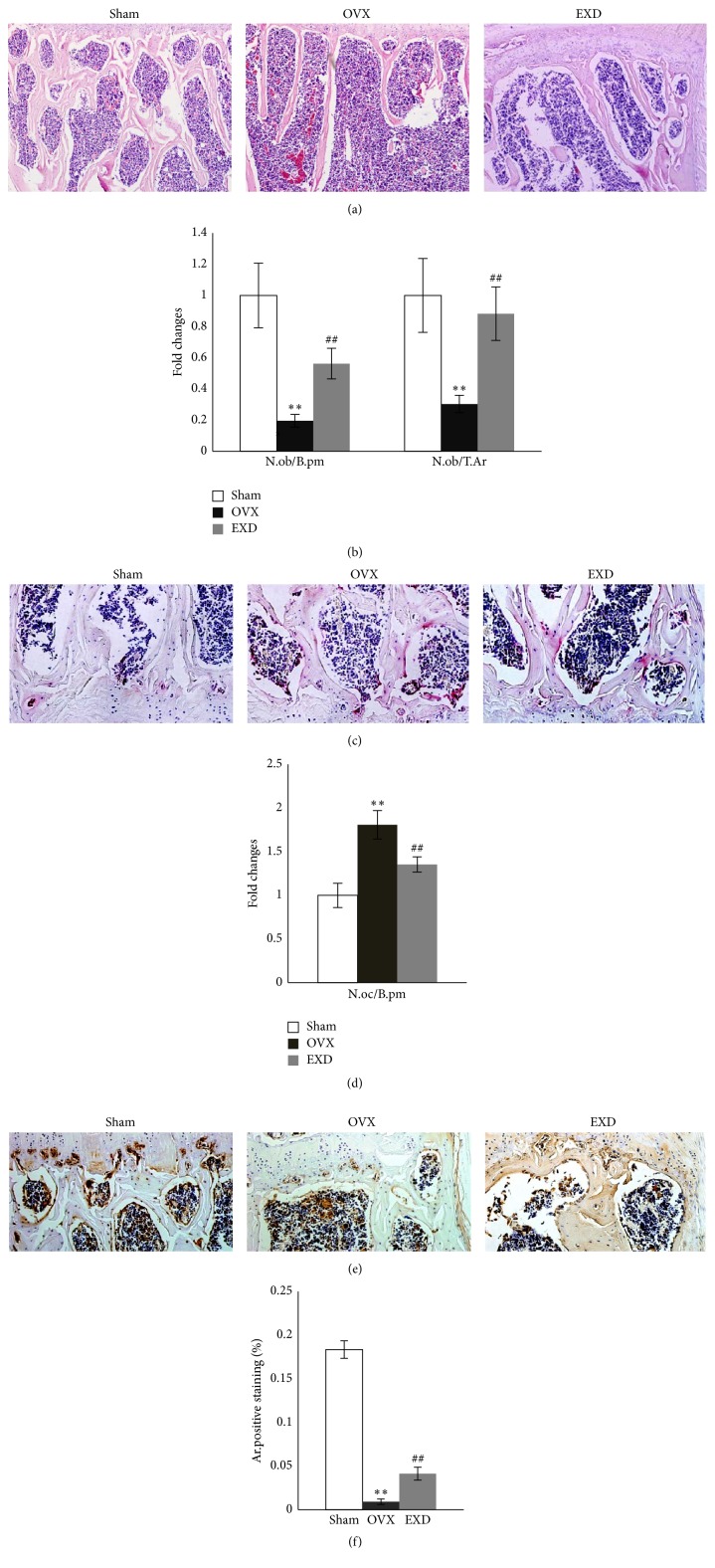
The effect of EXD treatment on activities of osteoblasts and osteoclasts in OVX mice. (a) Representative images of H & E staining demonstrated that OVX induced a loss of the number of bone trabeculae, which was reduced by EXD (×100). (b) Morphometric data displayed a great decrease of N.Ob/T.Ar and N.Ob/B.pm in OVX mice compared to Sham mice, which was partially rescued in EXD mice with 3-mon treatment. (c, d) Representative images of Trap staining showed EXD inhibited the elevated activity of osteoclasts induced by OVX as evaluated by N.Oc/B.pm (×200). (e) Immunostaining of L_4_ vertebrae with antibody against OC. Positive staining was indicated in brown (×200). (f) Quantitative data of (e). The columns represent the means ± SE *n* = 3 per group. ^*∗∗*^ *p* < 0.01 versus sham, ^##^ *p* < 0.01 versus OVX.

Then, the activities of osteoblasts and osteoclasts were evaluated by morphometry. Reduced trabecular numbers were observed in OVX mice compared to those in sham mice. EXD significantly inhibited this bone loss (Figure 2(a)). Quantification data indicated that the values of N.ob/T.Ar, N.ob/B.pm were decreased while N.oc/B.pm were increased in the OVX group compared to sham group (*p* < 0.01), indicating a decrease in the number of osteoblasts and an increase in the number of osteoclasts, which could be partially improved by EXD treatment (*p* < 0.01) (Figures 2(b)–2(d)).

Osteocalcin, secreted by osteoblasts and accumulated in the extracellular matrix of bone, is considered to be a marker of mature osteoblast [12, 13]. Positive staining for OC in bone marrow cells was decreased by OVX surgery, but EXD dramatically increased the number of OC positive cells in bone marrow (Figures 2(e) and 2(f)), suggesting EXD promotes osteoblastic differentiation.

### 3.3. Self-Renewal and Differentiation of bMSCs

**Figure 3:**
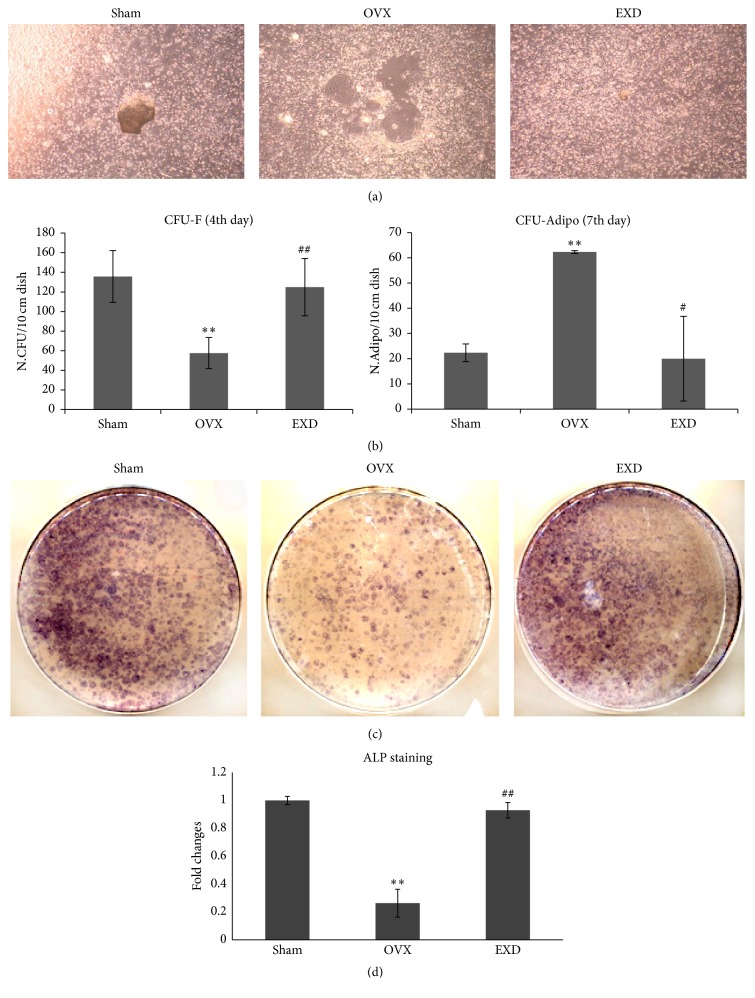
EXD treatment promotes self-renewal and osteoblastic differentiation of bMSCs from OVX mice. (a, b) BMSCs were harvested from mice of Sham, OVX and EXD groups and cultured for 4 or 7 days* ex vivo*. The results demonstrated that OVX reduced the number of spontaneously formed CFU-F but increased that of CFU-Adipo, indicating OVX induced adipogenic differentiation of bMSCs. EXD maintained the number of both CFU-F and CFU-Adipo as the similar level of sham mice (×120). (c, d) At the 7th day of culture, the ALP-positive staining of OVX bMSCs was decreased versus bMSCs from sham group. EXD dramatically increased the level of ALP-positive staining as compared to OVX group. The columns represent the means ± SE from three dishes (six mice) per group. ^*∗∗*^ *p* < 0.01 versus sham, ^#^ *p* < 0.05, ^##^ *p* < 0.01 versus OVX.

The results of CFU-F and CFU-adipo assay showed that EXD maintained CFU numbers of bMSCs, while decreased numbers of adipocyte differentiated from bMSCs compared to those from OVX mice (Figures 3(a) and 3(b)), indicating EXD enhanced bMSCs self-renewal capacity but impeded adipogenic differentiation.

ALP assay was done to determine whether EXD-treatment had any effect on the osteogenic differentiation of bMSCs. In the present study, EXD treatment dramatically increased the level of ALP activity of OVX mice compared to the nontreatment of OVX mice (Figures 3(c) and 3(d)).

### 3.4. Gene Expression Patterns and Common Pathways in bMSCs between* Ex Vivo* and* In Vitro* Experiments

**Figure 4:**
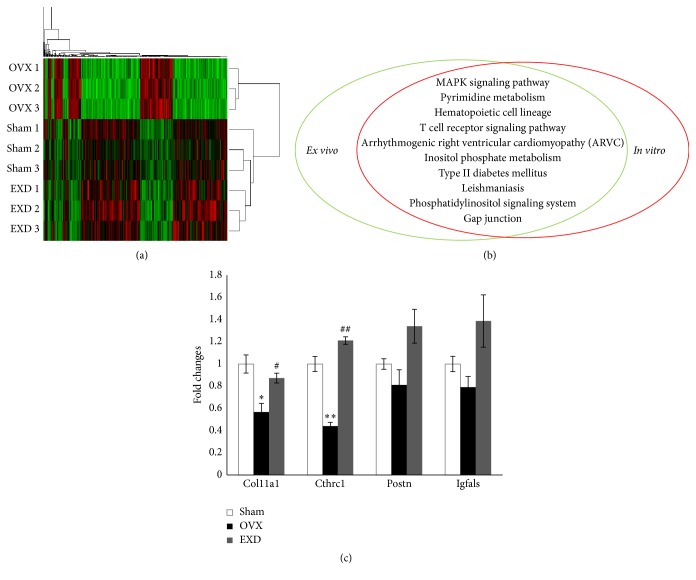
EXD treatment rescues 389 gene expressions involving in ten pathways both in* ex vivo* and* in vitro*. (a) Total 26991 genes were detected by microarray. Among them, 389 genes (a ratio of 1.44%) were fold changed > 1.5. These 389 genes were chosen for the hierarchical clustering and principal component analysis (PCA). The samples fell into three major classes, indicating the reliable qualities of microarray. The results showed EXD treatment in OVX mice shifted the gene expression profile toward the sham mice. (b) The pathways analysis demonstrated ten pathways overlapped between* ex vivo* and* in vitro* studies. (c) Quantification of qPCR results from* ex vivo* experiment. The columns represent the means ± SE (*n* = 3) per group. ^*∗*^ *p* < 0.05, ^*∗∗*^ *p* < 0.01 versus sham, ^#^ *p* < 0.05, ^##^ *p* < 0.01 versus OVX.

Total 389 genes (a ratio of 1.44% as compared to the total 26991 genes) that were fold changed > 1.5 (*p* < 0.01) among the Sham, OVX, and EXD groups were selected for clustering analysis. Results of the hierarchical clustering analysis were shown as a heat-map which can indicate the similarity in gene expression among either samples or genes. The samples fell into two major classes shown by the two branches of the dendrogram at the right of Figure 4(a). Interestingly, the classification corresponded to the experimental grouping, and the sham and EXD groups were grouped together while OVX groups were in the other pattern, suggesting a reliability of our gene expression measurement. The results manifested that EXD reversed the gene expression toward the Sham mice (Figure 4(a)).

The results of pathway analysis showed that 44 pathways were involved in EXD-treatment in* ex vivo* experiment while 83 pathways were in conditioned EXD-treat serum in* in vitro* experiment. Among them, 10 pathways overlapped including MAPK signaling pathway, pyrimidine metabolism, hematopoietic cell lineage, T cell receptor signaling pathway, arrhythmogenic right ventricular cardiomyopathy (ARVC), inositol phosphate metabolism, Type II diabetes mellitus, leishmaniasis, phosphatidylinositol signaling system, and gap junction (Figure 4(b)).

In a subsequent experiment, we chose four genes for qPCR verification: Col11a1 (type XI collage, alpha 1), Cthrc1 (Collage triple helix repeat containing 1), Postn (periostin, osteoblast specific factor), and Igfals (insulin-like growth factor binding protein, acid labile subunit). The effects of EXD on the expression levels of Col11a1 and Cthrc1 were verified while those of other two genes did not significantly altered (Figure 4(c)).

## 4. Discussion

EXD is commonly used in traditional Chinese clinic for relieving menopausal symptoms. It has been reported to exert an antiosteoporotic effect via promoting the activity of osteoblasts while inhibiting that of osteoclasts [8]. Our study demonstrated that EXD prevented OVX-induced bone loss based on both 2D and 3D bone histomorphometric analyses. Besides the effects on osteoblasts and osteoclasts, EXD stimulated self-renewal capacity and osteoblastic differentiation potential of bMSCs. It rescued several differentiated expressed genes of bMSCs by OVX injury toward their normal levels. The overlapped pathways have been elaborated which EXD acted on in* ex vivo* and* in vitro* studies. Our study first reported the proosteogenic effect of EXD on bMSCs and offered a figure of EXD's molecular targets of bMSCs.

By 3D bone histomorphometric results, we found series of bone mass indicators, such as BV/TV, Tb.N, Tb.Th, Tb.Sp, and Conn.D. were significantly corrected with EXD treatment in OVX mice, suggesting an active osteogenic process by EXD treatment [14]. The results were similar to Nian et al.'s report that the EXD suppressed the descent of bone mineral density after OVX surgery [3]. Moreover, the results of 3D histomorphometry analyses were consistent with those of 2D images, wherein we observed an increase in the activity of osteoblasts. In addition, the enhanced function of osteoblasts was evidenced by increased OC expression in the EXD-treated OVX mice.

Osteoblasts are considered to be originated from bMSCs. Some chemical constituents from EXD such as icariin and berberine have been reported to be potential to promote osteoblastic differentiation of bMSCs or osteoblasts [15]. As no conclusive evidence indicates unique markers of bMSCs [16], it hinders efforts to evaluate the direct function of bMSCs* in vivo*. So, we further observed the effects of EXD on the activities of bMSCs in* ex vivo* including their self-renewal capacity and potential of differentiation [17]. We determined that EXD-treatment markedly enhanced self-renewal capacity and the osteoblastic differentiation of bMSCs in the CFU and ALP assays, but reduced adipocytic differentiation, which was consistent with Xue's observation [18].

To further investigate the molecular mechanisms by which EXD exerts this effect, gene expression profiling studies were performed at 2 weeks after the OVX according to our previous study, and we determined a possible underlying mechanism of the activity of EXD on bMSCs [19]. In culture assay, we chose conditioned-serum because we want to mimic EXD status* in vivo* as well as its direct action on BMSCs not an indirect action such as on BMSCs niche. By combining these results with* ex vivo* data obtained from EXD-treated BMSCs* in vivo* (here, we had to expand BMSCs* ex vivo* to reach the numbers required for profiling), we would figure out the target genes in BMSCs regulated by EXD more accurately. The gene expression analysis showed EXD treatment clustered the gene expression pattern of EXD and Sham mice together, whereas it was separated from that of OVX model, suggesting a strong reversion of gene expression acted by EXD. EXD has been reported to reduce lipid peroxide (LPO), enhance superoxide dismutase (SOD), and catalase (CAT) gene expression levels in aging rats [20]. This decoction promoted the secretion of estradiol (E2) in granulosa cells to regulate the hypothalamus-pituitary-gonad (HPG) axis [21]. EXD regulated osteoblastic proliferation, activation, and apoptosis via increasing the expression of heat-shock protein 1, high mobility group protein (Hmgb1), acidic ribosomal phosphoprotein P0, histone 2, carbonyl reductase 1, ATP synthase, aldolase A, and Rho GDP dissociation inhibitor- (GDI-) alpha while decreasing carbonic anhydrase 3, prohibitin, hemiferrin, and far upstream element- (FUSE-) binding protein [8]. Among these EXD-regulated genes as reported, heat shock protein binding related genes Dnajc10, Dnajc13 (Accession numbers AK016269, AK147911) and histone cluster 1 encoding genes Hist1h1d, Hist1h2bg (Accession numbers BC119173, BC060304) were observed to be regulated by EXD in our gene profile results (Supplement 1, in Supplementary Material available online at http://dx.doi.org/10.1155/2016/4079210).

Several factors* in vivo* would influence the effect of drugs on bMSCs and caused differentiated expression of some undirected target genes. Meanwhile, factors* in vitro* such as culture conditions may change gene expression patterns acted by drugs. To better avoid the effects of these factors and to better observe the direct effect of EXD on bMSCs, the gene expression profiles were performed in both* in vivo* and* in vitro* studies. The overlapped pathways were analyzed. These pathways included MAPK signaling pathway, pyrimidine metabolism, hematopoietic cell lineage, T cell receptor signaling pathway, arrhythmogenic right ventricular cardiomyopathy (ARVC), inositol phosphate metabolism, Type II diabetes mellitus, leishmaniasis, phosphatidylinositol signaling system, and gap junction. Some of these pathways acted by EXD are directly related to bone metabolism. First, MAPK pathway as the upstream of bone morphogenetic protein-2 (BMP-2) [22, 23] plays a key role in osteoblast proliferation and differentiation. In skeletal development, activated MAPK contributes to stimulation of Runx2 phosphorylation and transcriptional activity [24]. Repression of MAPK pathway involves in PI3 K-induced osteoblast differentiation from MSCs [25]. Interestingly, the proosteogenic effect of berberine via Runx2 activation was related to p38 MAPK that was also essential for icariin-induced cardiomyocyte differentiation of murine embryonic stem cells [15, 26]. Second, hematopoietic cells and bMSCs are present in the bone marrow, and provide microenvironmental support for each other to control the others' function of proliferation, differentiation and migration [27–30]. Third, type II diabetes is possible to cause osteoporosis as a complication [31]. Forth, phosphatidylinositol family such as phosphatidylinositol 3-kinase (PI3-K) are active in stimulating bone formation, mediating osteoblast differentiation in human MSC through BMP-2, and interacting with MAPK signaling pathway [32–34]. Fifth, gap junction-mediated intercellular communication modulates BMP-2-induced chondrogenic differentiation or osteogenic differentiation of bMSCs [35, 36]. Although little has been reported of the other five pathways effects on bone development, EXD is possible to modulate activities of bMSCs indirectly via these pathways or to mediate other metabolic process which was presently reflected in the related genes expression of bMSCs as a hologram. Our qPCR results indicated EXD might exert an osteoprotective role by elevating collagen expressions.

## 5. Conclusion

Our results indicated that EXD exerts an osteoprotective effect in OVX-induced osteoporotic mice and elevates the self-renewal capacity the osteoblastic differentiation potential of bMSCs. The gene expression pattern is outlined of bMSC in EXD-treated OVX mice. EXD rescued several gene expressions that were dysregulated by OVX. These genes overlapped and are involved in ten pathways such as MAPK pathway, phosphatidylinositol signaling system, and gap junction between* ex vivo* and* in vitro* experiments. Our results contribute to further study of its molecular mechanism and traditional use in the treatment of postmenopausal osteoporosis.

## Supplementary Material

Supplement 1: Information of all genes whose expressions were changed (more than 1.5-fold) among Sham, OVX and EXD groups.

## Figures and Tables

**Table 1:** Primer information.

Gene	GenBank	Forward primer	Reverse primer	Product length
Postn	BC031449.1	ACGTCGTGGACAAACTCCTC	TGTTTCTCCACCTCCTGTGG	371
Col11a1	NM_007729.3	CGTCCCTTCTCTGCTAACCG	ACACAAGAGTGAATTGCAACCTG	900
Igfals	NM_008340.3	GCTCTGTACAAGGAACAATGGC	CCTGATACGATTGTGGCCGA	998
Cthrc1	NM_026778.2	CCAGGTCGGGATGGATTCAA	GGCAGGGACTGAAATCGTCA	518

## References

[1] Chen H. Y., Cho W. C. S., Sze S. C. W., Tong Y. (2008). Treatment of menopausal symptoms with Er-xian decoction: a systematic review. *The American Journal of Chinese Medicine*.

[2] Zhong L. L. D., Tong Y., Tang G. W. K. (2013). A randomized, double-blind, controlled trial of a Chinese herbal formula (Er-Xian decoction) for menopausal symptoms in Hong Kong perimenopausal women. *Menopause*.

[3] Nian H., Qin L.-P., Zhang Q.-Y., Zheng H.-C., Yu Y., Huang B.-K. (2006). Antiosteoporotic activity of Er-Xian Decoction, a traditional Chinese herbal formula, in ovariectomized rats. *Journal of Ethnopharmacology*.

[4] Qin L., Han T., Zhang Q. (2008). Antiosteoporotic chemical constituents from Er-Xian Decoction, a traditional Chinese herbal formula. *Journal of Ethnopharmacology*.

[5] Xue L., Wang Y., Jiang Y. (2012). Comparative effects of er-xian decoction, *Epimedium* herbs, and icariin with estrogen on bone and reproductive tissue in ovariectomized rats. *Evidence-Based Complementary and Alternative Medicine*.

[6] Wong K.-C., Lee K.-S., Luk H.-K. (2014). Er-xian decoction exerts estrogen-like osteoprotective effects in vivo and in vitro. *American Journal of Chinese Medicine*.

[7] Xue L., Jiao L., Wang Y. (2012). Effects and interaction of icariin, curculigoside, and berberine in Er-Xian decoction, a traditional Chinese medicinal formula, on osteoclastic bone resorption. *Evidence-Based Complementary and Alternative Medicine*.

[8] Zhu Z., Xue L.-M., Han T. (2010). Antiosteoporotic effects and proteomic characterization of the target and mechanism of an Er-Xian decoction on osteoblastic UMR-106 and osteoclasts induced from RAW264.7. *Molecules*.

[9] Rodríguez J. P., Garat S., Gajardo H., Pino A. M., Seitz G. (1999). Abnormal osteogenesis in osteoporotic patients is reflected by altered mesenchymal stem cells dynamics. *Journal of Cellular Biochemistry*.

[10] Bian Q., Liang Q.-Q., Hou W. (2011). Prolonged and repeated upright posture promotes bone formation in rat lumbar vertebrae. *Spine*.

[11] Bian Q., Huang J.-H., Liang Q.-Q. (2011). The osteogenetic effect of astragaloside IV with centrifugating pressure on the OCT-1 cells. *Pharmazie*.

[12] Bahlous A., Kalai E., Salah M. H., Bouzid K., Zerelli L. (2006). Biochemical markers of bone remodeling: recent data of their applications in managing postmenopausal osteoporosis. *Tunisie Medicale*.

[13] Wada S., Fukawa T., Kamiya S. (2007). Osteocalcin and bone. *Clinical Calcium*.

[14] Laffosse J.-M., Odent T., Accadbled F. (2010). Micro-computed tomography evaluation of vertebral end-plate trabecular bone changes in a porcine asymmetric vertebral tether. *Journal of Orthopaedic Research*.

[15] Lee H. W., Suh J. H., Kim H. (2008). Berberine promotes osteoblast differentiation by Runx2 activation with p38 MAPK. *Journal of Bone and Mineral Research*.

[16] Riekstina U., Cakstina I., Parfejevs V. (2009). Embryonic stem cell marker expression pattern in human mesenchymal stem cells derived from bone marrow, adipose tissue, heart and dermis. *Stem Cell Reviews and Reports*.

[17] Spitkovsky D., Hescheler J. (2008). Adult mesenchymal stromal stem cells for therapeutic applications. *Minimally Invasive Therapy and Allied Technologies*.

[18] Xue L., Wang Y., Liu L. (2011). A HNMR-based metabonomics study of postmenopausal osteoporosis and intervention effects of Er-Xian Decoction in ovariectomized rats. *International Journal of Molecular Sciences*.

[19] Bian Q., Liu S.-F., Huang J.-H. (2012). Oleanolic acid exerts an osteoprotective effect in ovariectomy-induced osteoporotic rats and stimulates the osteoblastic differentiation of bone mesenchymal stem cells in vitro. *Menopause*.

[20] Shen X. H., Fang Z. Q., Wu D. X. (1995). Effect of er-xian decoction and its disassembled prescription on enzyme activities and their gene expression of antioxidant enzymes in aging rat. *Zhongguo Zhong Xi Yi Jie He Za Zhi*.

[21] Dong B.-F., Fang Z.-Q., Shi J.-R. (2006). Effect of er xian decoction and its subdivisions on granulosa cells secretory function in rats. *Zhongguo Zhong Xi Yi Jie He Za Zhi*.

[22] Datta N. S., Kolailat R., Fite A., Pettway G., Abou-Samra A. B. (2010). Distinct roles for mitogen-activated protein kinase phosphatase-1 (MKP-1) and ERK-MAPK in PTH1R signaling during osteoblast proliferation and differentiation. *Cellular Signalling*.

[23] Ghosh-Choudhury N., Mandal C. C., Choudhury G. G. (2007). Statin-induced Ras activation integrates the phosphatidylinositol 3-kinase signal to Akt and MAPK for bone morphogenetic protein-2 expression in osteoblast differentiation. *The Journal of Biological Chemistry*.

[24] Ge C., Xiao G., Jiang D., Franceschi R. T. (2007). Critical role of the extracellular signal-regulated kinase-MAPK pathway in osteoblast differentiation and skeletal development. *Journal of Cell Biology*.

[25] Wu X., Chen S., Orlando S. A. (2011). p85*α* regulates osteoblast differentiation by cross-talking with the MAPK pathway. *The Journal of Biological Chemistry*.

[26] Ding L., Liang X.-G., Hu Y., Zhu D.-Y., Lou Y.-J. (2008). Involvement of p38MAPK and reactive oxygen species in icariin-induced cardiomyocyte differentiation of murine embryonic stem cells in vitro. *Stem Cells and Development*.

[27] De Barros A. P. D. N., Takiya C. M., Garzoni L. R. (2010). Osteoblasts and bone marrow mesenchymal stromal cells control hematopoietic stem cell migration and proliferation in 3D in vitro model. *PLoS ONE*.

[28] Liang X., Su Y.-P., Kong P.-Y. (2010). Human bone marrow mesenchymal stem cells expressing SDF-1 promote hematopoietic stem cell function of human mobilised peripheral blood CD34^+^ cells in vivo and in vitro. *International Journal of Radiation Biology*.

[29] Miura Y., Gao Z., Miura M. (2006). Mesenchymal stem cell-organized bone marrow elements: an alternative hematopoietic progenitor resource. *STEM CELLS*.

[30] Pontikoglou C., Deschaseaux F., Sensebé L., Papadaki H. A. (2011). Bone marrow mesenchymal stem cells: biological properties and their role in hematopoiesis and hematopoietic stem cell transplantation. *Stem Cell Reviews and Reports*.

[31] Isaia G., Bodrato L., Carlevatto V., Mussetta M., Salamano G., Molinatti G. M. (1987). Osteoporosis in type II diabetes. *Acta Diabetologica Latina*.

[32] Tang C.-H., Yang R.-S., Huang T.-H. (2006). Ultrasound stimulates cyclooxygenase-2 expression and increases bone formation through integrin, focal adhesion kinase, phosphatidylinositol 3-kinase, and Akt pathway in osteoblasts. *Molecular Pharmacology*.

[33] Osyczka A. M., Leboy P. S. (2005). Bone morphogenetic protein regulation of early osteoblast genes in human marrow stromal cells is mediated by extracellular signal-regulated kinase and phosphatidylinositol 3-kinase signaling. *Endocrinology*.

[34] Lee S.-U., Shin H. K., Min Y. K., Kim S. H. (2008). Emodin accelerates osteoblast differentiation through phosphatidylinositol 3-kinase activation and bone morphogenetic protein-2 gene expression. *International Immunopharmacology*.

[35] Zhang W., Green C., Stott N. S. (2002). Bone morphogenetic protein-2 modulation of chondrogenic differentiation in vitro involves gap junction-mediated intercellular communication. *Journal of Cellular Physiology*.

[36] Li X.-D., Chang B., Chen B. (2010). Panax notoginseng saponins potentiate osteogenesis of bone marrow stromal cells by modulating gap junction intercellular communication activities. *Cellular Physiology and Biochemistry*.

